# The forkhead transcription factor FOXM1 promotes endocrine resistance and invasiveness in estrogen receptor-positive breast cancer by expansion of stem-like cancer cells

**DOI:** 10.1186/s13058-014-0436-4

**Published:** 2014-09-12

**Authors:** Anna Bergamaschi, Zeynep Madak-Erdogan, Yu Jin Kim, Yoon-La Choi, Hailing Lu, Benita S Katzenellenbogen

**Affiliations:** 1 Department of Molecular and Integrative Physiology, University of Illinois at Urbana-Champaign, 524 Burrill Hall, Urbana, 61801 IL USA; 2 Laboratory of Cancer Genomics and Molecular Pathology, Samsung Biomedical Research Institute, Samsung Medical Center, Irwon-dong Gangnam-gu, Seoul, 135-710 Korea; 3 Department of Pathology, Samsung Medical Center, Sungkyunkwan University School of Medicine, Irwon-ro 81, Gangnam-gu, Seoul 135-710 Korea; 4 Department of Breast Cancer, Affiliated Tumor Hospital of Harbin Medical University, No.150 Haping Road, Nangag District Harbin, 150040 China

## Abstract

**Introduction:**

The forkhead transcription factor FOXM1 coordinates expression of cell cycle-related genes and plays a pivotal role in tumorigenesis and cancer progression. We previously showed that FOXM1 acts downstream of 14-3-3ζ signaling, the elevation of which correlates with a more aggressive tumor phenotype. However, the role that FOXM1 might play in engendering resistance to endocrine treatments in estrogen receptor-positive (ER+) patients when tumor FOXM1 is high has not been clearly defined yet.

**Methods:**

We analyzed FOXM1 protein expression by immunohistochemistry in 501 ER-positive breast cancers. We also mapped genome-wide FOXM1, extracellular signal-regulated kinase 2 and ERα binding events by chromatin immunoprecipitation followed by high-throughput sequencing (ChIP-seq) in hormone-sensitive and resistant breast cancer cells after tamoxifen treatment. These binding profiles were integrated with gene expression data derived from cells before and after FOXM1 knockdown to highlight specific FOXM1 transcriptional networks. We also modulated the levels of FOXM1 and newly discovered FOXM1-regulated genes and examined their impact on the cancer stem-like cell population and on cell invasiveness and resistance to endocrine treatments.

**Results:**

FOXM1 protein expression was high in 20% of the tumors, which correlated with significantly reduced survival in these patients (P = 0.003 by logrank Mantel-Cox test). ChIP-seq analyses revealed that FOXM1 binding sites were enriched at the transcription start site of genes involved in cell-cycle progression, maintenance of stem cell properties, and invasion and metastasis, all of which are associated with a poor prognosis in ERα-positive patients treated with tamoxifen. Integration of binding profiles with gene expression highlighted FOXM1 transcriptional networks controlling cell proliferation, stem cell properties, invasion and metastasis. Increased expression of FOXM1 was associated with an expansion of the cancer stem-like cell population and with increased cell invasiveness and resistance to endocrine treatments. Use of a selective FOXM1 inhibitor proved very effective in restoring endocrine therapy sensitivity and decreasing breast cancer aggressiveness.

**Conclusions:**

Collectively, our findings uncover novel roles for FOXM1 and FOXM1-regulated genes in promoting cancer stem-like cell properties and therapy resistance. They highlight the relevance of FOXM1 as a therapeutic target to be considered for reducing invasiveness and enhancing breast cancer response to endocrine treatments.

**Electronic supplementary material:**

The online version of this article (doi:10.1186/s13058-014-0436-4) contains supplementary material, which is available to authorized users.

## Introduction

Endocrine resistance in breast cancer is a process that appears to result from upregulation of growth factor and protein kinase signaling pathways that provide an alternate mechanism in support of tumor cell proliferation and survival [1]-[4]. Tamoxifen (TAM) has proven to be one of the most successful agents in the management of estrogen receptor-positive (ER+) breast cancers. When effective, it suppresses tumor growth and reduces the risk of relapse. Unfortunately, with time, about 50% of patients with ER+ breast cancer stop benefiting from TAM treatment and acquire resistance, leading to disease progression. Despite significant advances in defining some of the factors involved [5]-[8], the mechanisms underlying endocrine resistance are complex and not fully understood. Therefore, we have been interested in identifying and targeting, by inhibition or downregulation, key players that mediate endocrine resistance in ER+ breast cancer.

Many cancers are maintained in a hierarchical organization of rare cancer stem cells (CSCs) and more plentiful differentiated tumor cells. CSCs that are resistant to treatment not only have the capacity to give rise to differentiated tumor cells but also can lead to recurrence, metastasis and disease progression [9]-[11]. Therefore, endocrine resistance might be associated with the outgrowth of CSCs by promoting expansion of the CSC population or augmenting the production of key factors that regulate the CSC phenotype.

In our previous studies, we reported a correlation between overexpression of the protein 14-3-3ζ and early onset of recurrence in breast cancer patients [12]. We also uncovered a previously unknown relationship between 14-3-3ζ and FOXM1 in TAM resistance in breast cancer, with 14-3-3ζ acting upstream of FOXM1 to enhance the expression of FOXM1-regulated genes [13].

FOXM1 is a forkhead transcription factor that binds to chromatin and plays an important role in ERα signaling pathways [14]. FOXM1 is a key regulator of the cell cycle and is essential for formation of the mitotic spindle and correct chromosome segregation [15]. Its expression is very low in normal tissues, but elevated in many types of cancers [16]-[18]. High expression of FOXM1 is associated with a poor prognosis [19]-[22]. In addition to its role in mitosis and cytokinesis, this transcription factor regulates genes that control critical aspects of cancer, including differentiation [23], angiogenesis [24] and metastasis [16],[20].

In this study, we show that TAM-resistant (TamR) cells contain higher levels of FOXM1 than do parental cells sensitive to growth inhibition by TAM and that this is correlated with the presence of a larger CSC population. Further, in large cohorts of patient breast tumors that we examined, high *FOXM1* RNA and protein levels were found to correlate with a significantly faster onset of tumor recurrence and reduced overall survival. In cultured cells, FOXM1 promoted breast cancer aggressiveness and therapy resistance which could be reversed by FOXM1 inhibition or knockdown. Our genome-wide analyses using chromatin immunoprecipitation followed by high-throughput sequencing (ChIP-seq) revealed that TAM-specific FOXM1 binding sites are associated with genes encoding markers of CSCs and invasiveness and that overexpression of FOXM1 increases the proportion of CSCs and directly regulates the production of factors that promote aggressiveness and therapy resistance in breast cancer.

## Methods

### Cell culture, small interfering RNA, overexpression and ligand treatments

MCF-7 and T47D cells were obtained from the American Type Culture Collection (Manassas, VA, USA) and TamR MCF-7 cells (TamR cells) described previously [25] were cultured in minimal essential medium (MEM; Sigma-Aldrich, St Louis, MO, USA) supplemented with 5% calf serum (HyClone Laboratories, Logan, UT, USA), 100 μg/ml penicillin-streptomycin (Invitrogen, Carlsbad, CA, USA) and 25 μg/ml gentamicin (Invitrogen). Four days before control vehicle or ligand treatment, cells were seeded in phenol red-free MEM containing 5% charcoal-dextran-treated calf serum. Medium was changed on days 2 and 4 of culture before treatment. For three-dimensional cultures, 100 μl of Matrigel was spread in each well of a 12-well plate, and 8,000 cells were seeded and grown for 6 to 10 days. Spheroids were stained with Giemsa-Wright stain for 15 minutes at room temperature and washed twice with 1× phosphate-buffered saline (PBS) for 5 minutes each. Small interfering RNA (siRNA) experiments were carried out by transfecting 50 nM of siCtrl, siFOXM1 or siABCG2 from DharmaFECT reagent (Dharmacon, Lafayette, CO, USA) for 72 hours. Overexpression was performed as previously reported [12].

### ChIP and ChIP-reChIP assays

Cells were treated with 0.1% EtOH (vehicle) or 1 μM 4-hydroxytamoxifen (4-OH-TAM) for 45 minutes after pretreatment for 1 hour with the FOXM1-selective alternate reading frame (ARF) peptide inhibitor or mutant ARF control peptide [26] or with extracellular signal-regulated kinase kinase 1 (MEK1) inhibitor (AZD6244; Sellek Chemical, Houston, TX, USA) or control vehicle. After treatment, chromatin was cross-linked using 1% formaldehyde for 15 minutes at room temperature. Cells were washed with PBS, harvested and sonicated three times for 10 seconds in ChIP lysis buffer. Lysates were centrifuged for 10 minutes at 4°C. For immunoprecipitation of DNA-protein complexes, lysates were incubated overnight with antibodies to FOXM1 (GeneTex, Irvine, CA, USA) or extracellular signal-regulated kinase 2 (ERK2; Santa Cruz Biotechnology, Santa Cruz, CA, USA). Complexes were washed three times with radioimmunoprecipitation assay (RIPA) buffer (three times) and two times with Tris-EDTA (ethylenediaminetetraacetic acid). Following the overnight incubation at 65°C, ChIP DNA was isolated using a QIAGEN PCR purification kit (QIAGEN, Valencia, CA, USA) as per the manufacturer's suggestions. The DNA was used for ChIP-seq analysis and quantitative real-time PCR.

Sequential chromatin immunoprecipitation (ChIP-reChIP) experiments were done following the same ChIP protocol. After the first pull-down, immunoprecipitated material was recovered with 10 mM dithiothreitol in immunoprecipitation buffer at 37°C for 30 minutes, diluted and subjected to a second round of immunoprecipitation. Quantitative RT-PCR (qRT-PCR) was used to calculate recruitment to the regions studied, as described elsewhere [27].

### ChIP-seq and clustering analysis

For characterization of the FOXM1 and ERK2 cistromes from cells treated with 4-OH-TAM, the ChIP DNA was prepared into libraries according to Illumina Solexa ChIP-seq sample-processing methods (San Diego, CA, USA), and single-read sequencing was performed using the Illumina Solexa Genomic Analyzer using methods detailed previously [28]. Sequences generated were mapped uniquely onto the human genome (hg19) by Bowtie2 [29] with the default settings. A model-based analysis of ChIP-Seq algorithm [30] was used to identify enriched peak regions (default settings) with a *P*-value cutoff of 6.0E-7 and false discovery rate of 0.01. ChIP-seq data for FOXM1 and ERK2 binding sites are given as BED files in Additional file 1: Table S1. Cistrome data for ERα in MCF-7 cells treated with Tam are derived from a previous study [31].

The seqMINER density array method with a 300-bp window in both directions was used for the generation of clusters (that is, groups of loci having similar compositional features) [32]. This ChIP-seq data interpretation platform allows the comparison and integration of multiple ChIP-seq data sets and their extraction and visualization of specific patterns as described previously [28]. BED files for each cluster were used for further analysis with Galaxy Cistrome integrative analysis tools (Venn diagram, conservation, Cis-regulatory Element Annotation System (CEAS)) [33].

### Motif and Gene Ontology category analysis

Overrepresented Gene Ontology (GO) biological processes were determined by utilizing the web-based DAVID Bioinformatics Resources database [34],[35], GeneSpring and web-based GREAT (Genomic Regions Enrichment of Annotations Tool) software [36]. Motif enrichment analysis was done using SeqPos [33]. Conservation of the binding sites was determined using web-based CEAS software of the Cistrome/Galaxy platform [37]. Default parameters were used in all software.

### RT-PCR and quantitative PCR

Total RNA was isolated from cells using TRIzol reagent (Invitrogen). RNA samples were reverse-transcribed using SuperScript II reverse transcriptase (Invitrogen), and RT-PCR was carried out on the ABI Prism 7900HT Sequence Detection System using SYBR Green PCR Master Mix (Applied Biosystems, Foster City, CA, USA) as described previously [38]. Primer sequences for the genes studied were obtained from the Harvard Primer Bank [39]. Sequences are available on their website.

### Microarray gene expression data analysis and statistics

Total RNA was used to generate complementary RNA (cRNA), which was labeled with biotin according to protocols recommended by Affymetrix (Santa Clara, CA, USA). All analyses were done using three or more samples for each treatment. The biotin-labeled cRNA was hybridized to Affymetrix U133 plus 2.0 GeneChips, which contain oligonucleotide probe sets for over 47,000 transcripts. After being washed, the chips were scanned and analyzed using Affymetrix processing software. All microarray gene expression data have been deposited in the Gene Expression Omnibus database [GEO:GSE55204]. CEL files were processed using GeneSpring GX 11.0 software (Agilent Technologies, Santa Clara, CA, USA) to obtain fold changes and *P*-values with the Benjamini and Hochberg multiple-test correction [40] for each gene for TAM treatment relative to the vehicle control in each cell background. We considered genes with fold changes greater than two and *P*-values <0.05 as statistically significant and differentially expressed. For analyses of microarray data sets from TAM- treated breast cancer patients, we used Frasor *et al*. data [GEO:GSE1379] [38] and Buffa *et al*. data [GEO:GSE2221] [41]. Multifactor analysis was computed in WinSTAT statistics add-in for Excel software (R. Fitch software). Differences between two groups were assessed using an unpaired t-test. Data involving more than two groups were assessed by analysis of variance with Dunnett's multiple-comparisons test. Differences were considered significant at *P* < 0.05. Additional statistical analyses done are indicated in the figure legends.

### Western blot analysis

Whole-cell extracts were prepared using 1× RIPA lysis buffer (Upstate/Chemicon, Billerica, MA, USA) supplemented with 1× cOmplete Protease Inhibitor Cocktail mixture (Roche Applied Science, Basel, Switzerland). Proteins were separated on 4% to 20% gradient SDS-PAGE gels and transferred to nitrocellulose membranes. For Western blot analysis, we used antibodies against FOXM1, ERK1 and ERK2 (Santa Cruz Biotechnology), β-actin (Sigma-Aldrich), phosphorylated mitogen-activated protein kinase (pMAPK) (Cell Signaling Technology, Danvers, MA, USA) and CD44 (BD Biosciences, San Jose, CA, USA).

### Cell proliferation assay

A WST-1 assay (Roche Applied Science) was used to quantify cell viability. Absorbance was read at 450 nm on a PerkinElmer Victor X Multilabel Plate Reader (PerkinElmer, Waltham, MA, USA), and all assays were performed in triplicate as described elsewhere [13],[42].

### Fluorescence-activated cell sorting and immunofluorescence

For fluorescence-activated cell sorting (FACS), cells were detached and then stained with antibodies for CD44, CD24, ABCG2 (BD Biosciences and Cell Signaling Technology) at 1:100 dilution in PBS containing 1% fetal calf serum. FACS-sorted cells were collected into cell medium for plating or into RNA*later*™ buffer for RNA extraction. To test for ABCG2+ activity, 1 × 10^6^ cells were incubated with 5 μM Hoechst 33258 dye at 37°C for 90 minutes. All samples were analyzed and sorted using a FACSAria III instrument (BD Biosciences).

### Invasion assay

Breast cancer cells were seeded on precoated filters (8-μm pore size) after membrane rehydration (BD Biosciences). Following incubation for 48 hours at 37°C, cells were fixed in 10% formalin buffer and stained using crystal violet. Noninvasive cells on the surface of the filter were removed using a cotton swab. Invasion was quantified by determining the percentage of cells that had invaded the filter compared to the total number seeded as described previously [13],[42].

### Breast tumor cohort and FOXMimmunohistochemistry and statistical analysis

A tissue microarray (TMA) from the Samsung Medical Center Breast Cancer Biomarker Study was utilized for the analysis of FOXM1 status. Detailed clinical features and molecular subtype classification have been reported elsewhere [43],[44]. Briefly, from among 815 tumors, 501 were assigned as ERα-positive and used for the immunohistochemical detection of FOXM1 expression. TMA sections were incubated for 1 hour at room temperature with mouse anti-human FOXM1 antibody (ab55006; Abcam, Cambridge, MA, USA) diluted 1:400. The detection system EnVision+ for mouse antibody (K4001; Dako, Glostrup, Denmark) was applied according to the manufacturer's instructions. Slides were stained with liquid diaminobenzidine tetrahydrochloride (DAB+), a high-sensitivity substrate chromogen system (K3468; Dako). Counterstaining was performed with Mayer's hematoxylin. FOXM1 expression was scored using a semiquantitative method based on the following four classes: score 0 (no staining or nuclei staining observed in <10% of the tumor cells), score 1+ (faint nuclear staining detectable in >10% of the tumor cells), score 2+ (weak to moderate nuclear staining observed in >10% of the tumor cells) and score 3+ (strong nuclear staining observed in >30% of the tumor cells). Representative photomicrographs of each of the scoring categories are shown in Additional file 2: Figure S4. Patients with tumor scores ranging from 0 to 1 were classified as FOXM1-negative/low expression, and those who had scores of 2+ and 3+ were classified as FOXM1-high expression group. Disease-free survival was defined as the time from the date of diagnosis to the date of documented relapse, including locoregional recurrence and distant metastasis. Survival curves were constructed using the Kaplan-Meier method, and the logrank test was used to compare the mean survival rates across the groups. The logrank test with Bonferroni's correction was used for the subgroup survival analysis.

### Accession numbers and data availability

Gene expression data are available in the GEO database [GEO:GSE55204]. ChIP-Seq data files for FOXM1 and ERK2 binding sites in TAM-treated cells are given as BED files in Additional file 1: Table S1.

## Results

### Estrogen receptor-positive breast tumors with high expression of FOXM1 show early time to recurrence, and tumors positive for both 14-3-3ζ and FOXM1 show earliest time to recurrence

**Figure 1:**
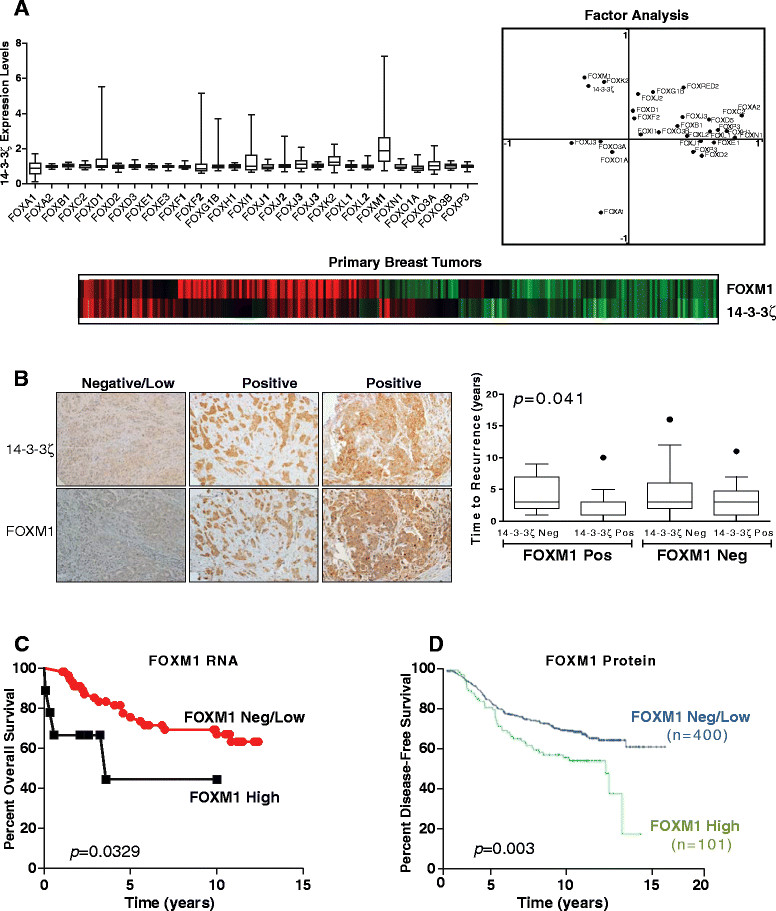
**Clinical data indicating that high expression of FOXM1 in estrogen receptor-positive breast tumors is correlated with high expression of 14-3-3ζ and a poor clinical outcome. (A)** Among the FOX family members, FOXM1 expression level most highly correlated with 14-3-3ζ expression level in primary breast tumors. Microarray gene expression data are derived from our findings in 251 estrogen receptor-positive (ER+) breast tumors [38]. Box plots are shown for 27 FOX family members with error bars spanning minimum to maximum values, and heat maps (below) show strong agreement between expression of FOXM1 and 14-3-3ζ/YWHAZ in breast tumors. Factor analysis (top left) reveals that FOXM1 and 14-3-3ζ are linearly correlated. **(B)** Tumors positive for 14-3-3ζ and FOXM1 by IUC show the earliest time to recurrence. *P* = 0.041 based on two-way analysis of variance with Dunnett's multiple-comparisons test for multigroup comparison. **(C)** Kaplan-Meier stratification of overall survival of tamoxifen-treated patients [38],[41] based on FOXM1 mRNA expression. **(D)** Kaplan-Meier analysis of disease-free survival of ER+ patients according to IHC scores for negative/low FOXM1 expression (blue curve) versus high FOXM1 expression (green curve) (*P* = 0.003) analyzed using the logrank test.

We reported previously that women with breast tumors expressing high levels of the scaffold adaptor protein 14-3-3ζ had a poor prognosis [12],[13]. We also observed by molecular analyses that FOXM1 was regulated by 14-3-3ζ and was downstream of 14-3-3ζ [12]. Because *FOXM1* is a transcription factor that might regulate the expression of genes that engender this less good patient outcome, we first investigated the relationship between *FOXM1* and *14-3-3ζ*. As shown in Figure 1A, we examined the mRNA expression of 27 forkhead transcription factors in 251 primary ERα-positive breast tumors. Notably, we observed that expression of *14-3-3ζ* in these tumors was most highly correlated with expression of *FOXM1* (*r* = 0.59, *P* = 9.03E-13) and next with *FOXK2* expression (*r* = 0.38, *P* = 2.07E-10) (Figure 1A). This good correlation in expression of *FOXM1* and *14-3-3ζ/YWHAZ* can be seen in the gene expression heat map and in the factor analysis plot (Figure 1A). Furthermore, tumors positive for both *14-3-3ζ* and *FOXM1* showed the earliest time to recurrence (*P* = 0.041) (Figure 1B). Of interest, analysis of our microarray gene expression data from a large study with TAM-treated breast cancer patients [38] showed that high *FOXM1* mRNA expression in tumors was associated with a less good patient survival (Figure 1C). Likewise, our immunohistochemistry (IHC) analysis of tumors from a cohort of 501 ERα-positive breast tumors revealed that high expression of FOXM1 protein was associated with a much poorer patient survival (Figure 1D). Kaplan-Meier logrank survival analysis showed that time to recurrence was significantly longer in patients with tumors negative or low for FOXM1 compared to patients with tumors with high FOXM1 protein (IHC score 2 or 3), which represented about 20% of all ER+ tumors (*P* = 0.003) (Figure 1D). FOXM1 multivariate Cox regression analysis also revealed FOXM1 to have a significant *P*-value (*P* = 0.0048, odds ratio = 1.661, 95% confidence interval = 1.177 to 2.343) when FOXM1 was stratified by recurrence-free survival. Thus, high *FOXM1* mRNA or protein confers a worse prognosis in ER+ breast cancers.

### FOXMis elevated in tamoxifen-resistant breast cancer cells and contributes to the endocrine-resistant phenotype

**Figure 2:**
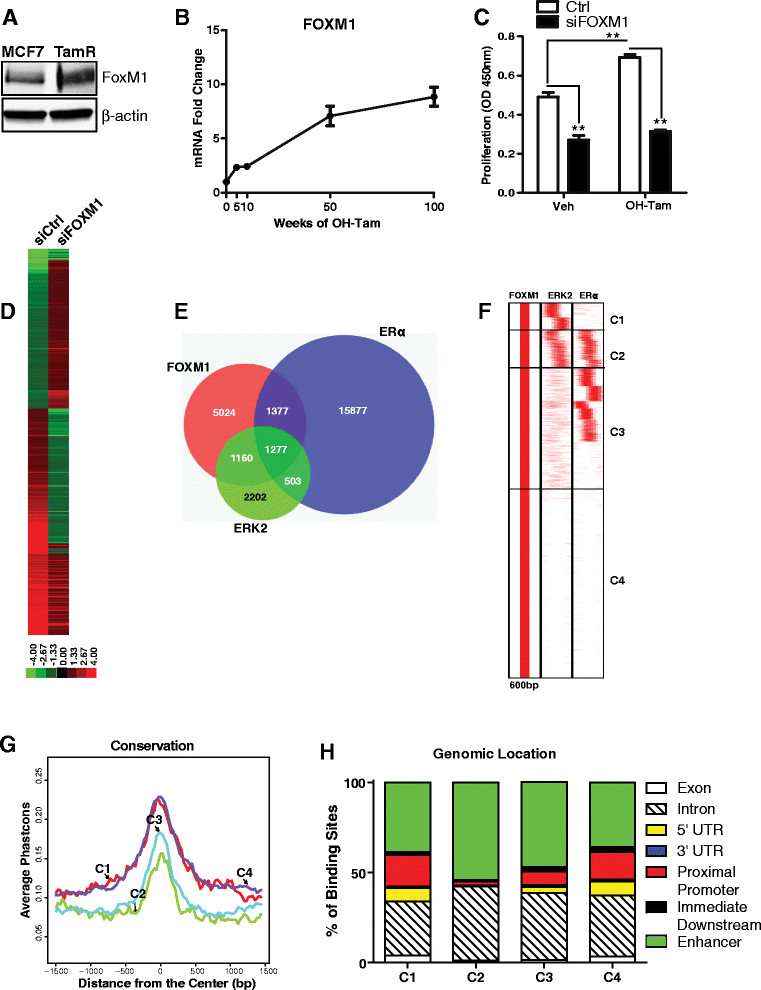
**FOXM1 is elevated by tamoxifen treatment and genome-wide analysis of FOXM1, ERK2 and ERα chromatin binding sites by ChIP-seq after tamoxifen treatment, and gene expression profiling, and clustering analyses. (A)** FOXM1 protein levels in MCF-7 and tamoxifen-resistant (TamR) cells monitored by Western blot analysis. **(B)** mRNA levels of FOXM1 mRNA over time of 1 μM 4-hydroxytamoxifen (OH-TAM) exposure. The fold change in FOXM1 gene expression in the presence of tamoxifen- over vehicle-treated cells was calculated using the comparative threshold cycle method, with the ribosomal protein 36B4 mRNA used as an internal control. **(C)** Proliferation of TamR control cells (Ctrl) and cells with FOXM1 knockdown (siFOXM1). OD, Optical density. Data are mean ± SEM. **P < 0.01. **(D)** Heat map representing the expression levels of OH-Tam-regulated genes in Ctrl and siFOXM1 MCF-7 cells treated with control vehicle or OH-Tam. Heat map shows fold change for gene expression in Tam-treated vs. vehicle-treated cells with or without siFOXM1. **(E)** Venn diagram showing overlap of FOXM1, extracellular signal-regulated kinase 2 (ERK2) and estrogen receptor α (ERα) chromatin binding sites in cells after 45 minutes of OH-Tam treatment and chromatin immunoprecipitation followed by high-throughput sequencing (ChIP-seq) analysis. FOXM1-Tam and ERK2-Tam cistrome data are from this study; the ERα-Tam cistrome data are derived from Hurtado *et al*. [31]. **(F)** Clustering of the binding sites for FOXM1, ERK2 and ERα after cell treatment with Tam using seqMINER software based on co-occupancy of the different factors within a 600-bp window. **(G)** Conservation of clusters C1, C2, C3 and C4 binding sites among vertebrates. **(H)** Genomic location of clusters C1 to C4 binding sites identified by using the web-based CEAS tool. UTR, Untranslated region.

On the basis of these clinical observations, we examined the levels of FOXM1 in MCF-7 parental and TamR cells and found threefold higher levels of FOXM1 protein in TamR cells (Figure 2A). We also monitored the kinetics of increase of FOXM1 over time of 4-OH-TAM exposure and observed a progressive and large (approximately tenfold) increase in *FOXM1* mRNA over the course of 100 weeks examined (Figure 2B). Moreover, proliferation of control vehicle- or 4-OH-TAM treated cells was reduced by FOXM1 siRNA (Figure 2C), which resulted in almost full loss of FOXM1 protein. The TamR cells were growth-stimulated by 4-OH-TAM (Figure 2C), and this stimulation was eliminated, and cell proliferation decreased, when FOXM1 was knocked down, indicating that FOXM1 plays a role in the resistance to TAM (Figure 2C).

To assess how FOXM1 might affect gene regulation by 4-OH-TAM, we performed Affymetrix gene expression microarray analysis on MCF-7 cells treated with 4-OH-TAM with (siFOXM1) and without (siCtrl) knockdown of FOXM1. Using a fold change greater than two and a *P*-value <0.05, we found 546 genes to be differentially expressed (Figure 2D). GO analysis of the functional annotations of the differentially regulated genes revealed an enrichment for cell cycle and chemotaxis categories in genes downregulated upon knockdown of FOXM1 in 4-OH-TAM-treated cells vs. control, whereas apoptosis and programmed cell death genes were upregulated and enriched when FOXM1 was decreased by siFOXM1 treatment.

### Genome-wide analysis of FOXM1 chromatin binding by ChIP-seq, gene regulation by FOXM1 and clustering of binding sites and delineation of gene functional categories

Next, we undertook the genome-wide characterization of FOXM1 binding sites by ChIP-seq analysis to address how TAM treatment affected the recruitment of FOXM1 to specific genomic loci. In our FOXM1 cistrome from MCF-7 cells treated with TAM, we observed that 22% of the FOXM1 binding sites were also shared by ERα binding sites after TAM treatment as reported by Hurtado *et al*. [31] (Figure 2E). Hence, FOXM1 also bound to a significant number of unique sites, suggesting that FOXM1 might uniquely control the transcription of specific sets of genes in a manner independent from ERα or that ERα might operate along with FOXM1 present at different sites via looping together of different chromatin locations.

A prominent feature of acquired TAM resistance is the hyperactivation of MAPK. In light of this and the fact that we have previously shown ERK2 to be recruited to chromatin by ERα after estradiol treatment of breast cancer cells [45], we assessed the recruitment of ERK2 to chromatin after 4-OH-TAM exposure of cells and the extent of overlap of ERK2 binding with FOXM1 and ERα binding (Figure 2E). ERK2 and FOXM1 co-occupied more sites (47% of ERK2 sites) than ERK2 and ERα (35% of ERK2 sites), suggesting that FOXM1 becomes a major transcription factor driving ERK2 to the chromatin in the presence of 4-OH-TAM.

To obtain a better picture of the chromatin binding landscape of these factors, we utilized a clustering approach using seqMINER [32], which compares the presence of multiple factors at a given chromosomal location within a 600-bp window and clusters together those binding sites that share a similar pattern of factor localization. We have previously shown, through this type of cluster analysis, that binding sites can be classified based on a series of factor recruitments, enabling the highlighting of commonalities in regulatory modes for modulated genes in each specific cluster [28].

Similarity in the composition of binding sites for FOXM1, ERK2 and ERα and directionality of gene regulation marked classes of genes that are part of the same functional category, denoted as clusters C1 to C4 (Figure 2F). We further characterized these clusters based on GO enrichment, transcription factor prediction using SeqPos, genomic distribution and binding site conservation among species using CEAS. Cluster 1 (C1) was represented by binding sites occupied by FOXM1 and ERK2 and was enriched in genes involved in stem cell development, cell-cycle G_2_-M-related genes and transforming growth factor β, platelet-derived growth factor and hypoxia-inducible factor 2α (HIF2α) signaling pathways (Table 1). FOXM1 and GATA binding motifs were significantly enriched in this cluster. Further, C1 and C4 had the highest sequence conservation among species (Figure 2G) and had a substantial presence of binding sites at proximal promoter genomic locations (Figure 2H, red), with clusters C2 and C3 showing the greatest proportion of binding sites at enhancer and intronic regions.

**Table 1:** **Enriched Gene Ontology functions, pathways and transcription factor motifs in the seqMINER-identified FOXM1 binding site clusters** ^**a**^

	Enriched GO functions and pathways	Enriched TFs
**Cluster 1**	• Cell-cycle G_2_-M	FOXM1, GATA
	• Stem cell development	
	• Increased adenoma	
	• TGF-β, PDGF and HIF2α signaling pathways	
**Cluster 2**	• Genes regulated by ESR1	ERα, FOXM1, CREB, ATF3
	• Genes upregulated in the luminal B subtype of breast cancer	
**Cluster 3**	Subgroup A	
	• Genes associated with acquired endocrine therapy resistance in breast tumors expressing ESR1	ERα, FOXM1, AP-1
	• Focal adhesion	
	• Neoplasm	
	Subgroup B	FOXM1, ERα, AP-1
	• Cell substrate adherens junction	
	• Cytoskeleton regulation and rearrangement	
	• Neoplasm	
	• Response to hypoxia	
	Subgroup C	FOXM1, GATA, ERα
	• Epithelial cell development	
	• p53 pathway	
	• HIF1α transcription factor network	
**Cluster 4**	• Translation	FOXM1, GATA, Elk, AP-1, JunD
	• Mammary gland hyperplasia	
	• Abnormal apoptosis	
	• Abnormal mitotic index	

^a^AP-1, Activator protein 1; ATF3, Activating transcription factor 3; CREB, cAMP response element-binding-protein; ERα, Estrogen receptor α; GO, Gene Ontology; HIF, Hypoxia-inducible factor; PDGF, Platelet-derived growth factor; TFs, Transcription factors; TGF-β, Transforming growth factor β.

Cluster C2 (Figure 2F) was represented by binding sites containing ERα, FOXM1 and ERK2. Genes harboring these binding sites were classified as ERα-regulated genes that belonged to the luminal B breast cancer subtype (Table 1). These binding sites were mainly localized at enhancers (Figure 2H) and were enriched for ERα, FOXM1, CREB and ATF3 binding motifs. Cluster C3 was also characterized by occupancy by FOXM1 and ERα, but not ERK2, and was associated with genes expressed in endocrine-resistant cells and genes associated with cytoskeletal regulation, focal adhesion, epithelial cell development, the p53 pathway and the HIF1α network (Table 1). C4 binding sites were mainly enriched in genes involved in translation, mammary gland hyperplasia and abnormal apoptosis and mitotic index (Table 1). FOXM1, GATA, Elk, activator protein 1 and JunD motifs were enriched at these sites. In Additional file 2: Figure S1, we also present for comparison the binding site clustering pattern we obtained for ERα in MCF-7 cells after treatment with control vehicle, TAM or estradiol (E2) to explore if FOXM1 and ERα co-occupy chromatin sites in the presence of E2; this divided C3 into three subgroups (Additional file 2: Figure S1). This analysis suggests that FOXM1 might act as a pioneering factor for ERα binding in the presence of TAM and E2. The cluster patterns indicate that FOXM1 has specific (C1 and C4) and common binding sites with ERα (C2 and C3) and highlight the ERα-dependent and -independent roles of FOXM1 in the breast cancer phenotype.

We compared the FOXM1 binding sites from our study done in cells treated with 10^−6^ M 4-OH-TAM with those described in the only other report on FOXM1 binding sites in MCF-7 cells, from Sanders *et al*. [14] (Additional file 2: Figure S2). In that study, these binding sites were examined in fetal bovine serum with no added hormone or hormone antagonist. Of the FOXM1 binding sites we identified in TAM-treated cells, 55% were also found in the Sanders *et al*. study. Many FOXM1 chromatin binding sites differed, however, no doubt reflecting the very different cell treatment conditions.

### Cluster genes can discriminate between patient breast tumors with different clinical outcomes

**Figure 3:**
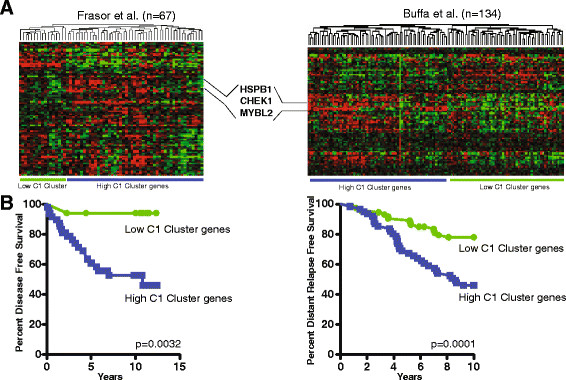
**Expression of FOXM1-regulated genes in cluster C1 stratify breast cancer patients based on outcomes.** **(A)** Heat map of hierarchical clustering of the expression of FOXM1-regulated C1 genes in two independent cohorts of patients treated with tamoxifen. Frasor *et al.*[38], Buffa *et al.*[41]. **(B)** Kaplan-Meier stratification of samples based on expression and hierarchical clustering of C1 genes.

Genes within 20 kb of binding sites belonging to cluster C1 and whose expression was impacted by FOXM1 knockdown were used to generate a gene predictor that was employed to interrogate two large, independent data sets [38],[41] of ERα-positive breast cancer patients treated with TAM. Hierarchical clustering was used to stratify patients according to the expression of this C1 gene signature. As shown in Figures 3A and 3B, the C1 signature very effectively stratified the patients based on clinical outcome, indicating that FOXM1 regulatory sites and associated target genes may play a pivotal role in tumor progression and TAM resistance. Of interest, this signature included the genes *HSPB1, CHEK1* and *MYBL2/B-MYB*, all of which have functions known to be associated with a poor prognosis [46]-[48].

### FOXM1 recruitment to C1 chromatin binding sites and impact of FOXM1 and extracellular signal-regulated kinase 2 inhibition

**Figure 4:**
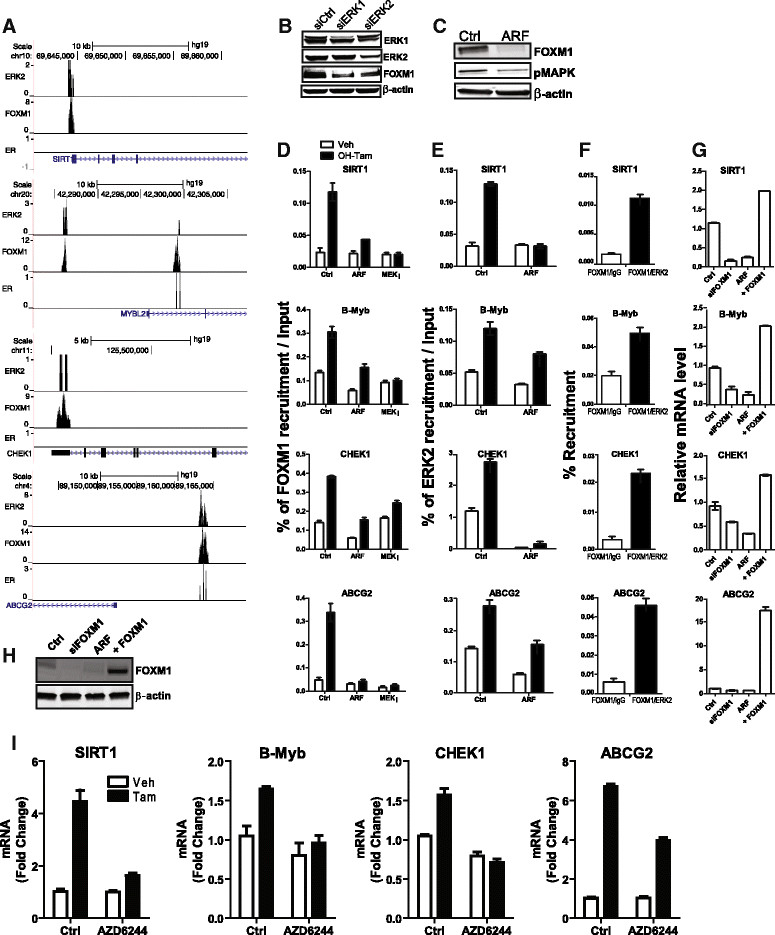
**FOXM1 and ERK2 co-occupy genomic loci of cluster C1 genes, and impact of knockdown of FOXM1, or ERK1, or ERK2, or treatment with FOXM1 inhibitor. (A)** UCSC Browser location of the binding sites we identified for FOXM1, estrogen receptor α and extracellular signal-regulated kinase 2 (ERK2) for four representative genes in the C1 cluster (SIRT1*,* B-Myb*,* CHEK1 and ABCG2). **(B)** Western blot showing ERK1, ERK2 and FOXM1 levels after small interfering RNA (siRNA) knockdown of ERK1 or ERK2. **(C)** Western blot showing FOXM1 and phosphorylated mitogen-activated protein kinase levels after alternate reading frame (ARF) (FOXM1 inhibitor) treatment. **(D)** FOXM1 recruitment to chromatin sites was assessed after ARF or extracellular signal-regulated kinase kinase 1 (MEK1) inhibitor treatment and followed by quantitative RT-PCR (qRT-PCR). **(E)** Chromatin immunoprecipitation (ChIP) for ERK2 was assessed after ARF or control vehicle treatment. **(F)** ChIP-reChIP showing binding site co-occupancy by FOXM1 and ERK2. Immunoprecipitation was done first for FOXM1 and then for ERK2. **(G)** Levels of representative C1 genes in siCtrl- or siFOXM1-treated cells or in cells with ARF treatment or overexpression of FOXM1. **(H)** Western blot showing FOXM1 levels after siRNA or ARF treatment or overexpression. **(I)** MEK1 inhibitor blocks tamoxifen (TAM) stimulation of FOXM1 target genes. Cells were pretreated with 10 μM MEK1 (AZD6244) or vehicle for 45 minutes and then treated with vehicle (0.1% EtOH) or 1 μM TAM in the presence or absence of inhibitor for 4 hours. RNA was isolated and qPCR analysis was done.

To investigate the relationship between FOXM1 and ERK2 at sites of cobinding in cluster C1, we examined the recruitment of FOXM1 and ERK2 following the treatment of MCF-7 cells with 4-OH-TAM or vehicle in cells treated or not with a FOXM1-specific inhibitor (p19^ARF^ 26–44 peptide) [49] or with a MEK1 inhibitor (AZD6244). We performed ChIP followed by qPCR for regions where both FOXM1 and ERK2 were identified in four of these genes: *SIRT1, MYBL2, CHEK1* and *ABCG2* (Figure 4A). By Western blot analysis, we show the effect of ERK1 and ERK2 knockdown on the levels of FOXM1 (Figure 4B) and the effect of FOXM1 inhibition by ARF on the levels of pMAPK (Figure 4C). Inhibition of FOXM1 by ARF or inhibition of ERK2 activation by MEK1 using a MEK1 inhibitor significantly decreased the recruitment of FOXM1 after 4-OH-TAM treatment to all four genes tested (Figure 4D). We also performed ChIP for ERK2 target genes before and after ARF treatment, and we observed a significant reduction in the recruitment of ERK2 to target gene loci after FOXM1 inhibitor treatment (Figure 4E). Moreover, the results of ChIP-reChIP experiments (Figure 4F) indicated that FOXM1 and ERK2 co-occupy the investigated genomic loci. We also modulated FOXM1 downward by inhibition (ARF) or by knockdown (siRNA) or upward by overexpression, and then examined effects on the mRNA levels (Figure 4G) of genes harboring these binding sites within a 20-kb window and on FOXM1 protein levels (Figure 4H). We observed that FOXM1 was crucial for the expression of *SIRT1, MYBL2, CHEK1* and *ASCG2* (Figure 4G). In addition, MEK1 inhibitor AZD6244 blocked TAM stimulation of these FOXM1 target genes (Figure 4I). These findings suggest that the binding of both FOXM1 and ERK2 to these chromatin binding sites is required for expression of these genes. Of note, transient knockdown of ERK1 or ERK2 significantly decreased the cellular level of FOXM1 and inhibition of FOXM1 also decreased the level of pMAPK, suggesting that they are part of an interdependent regulatory loop.

### FOXMtranscription program drives expansion of cancer stem-like cells

**Figure 5:**
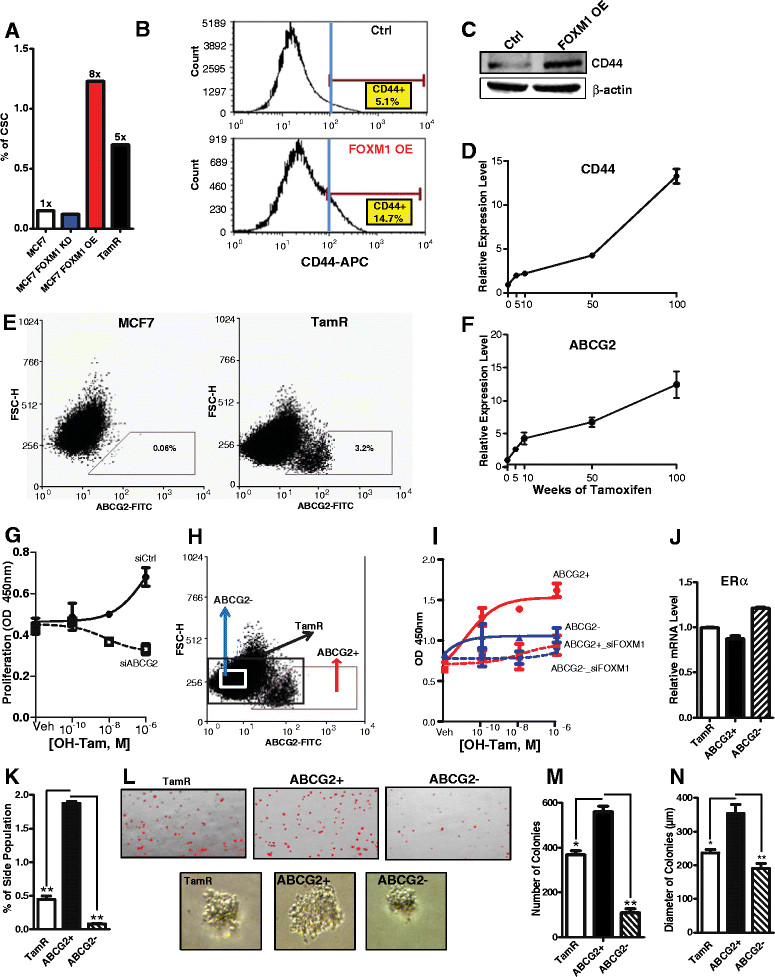
**FOXM1 increases expression of cancer stem cell markers and colony formation and invasiveness. (A)** FOXM1 levels impact the percentage of the CD44^+/CD24−/low^ population in MCF-7 cells. CSC, Cancer stem cell-like cells; KD, Knockdown; OE, Overexpression; TamR, Tamoxifen-resistant cells. **(B)** Increased levels of the marker CD44 by fluorescence-activated cell sorting (FACS) analysis. FACS analyses were done in three separate experiments, and a representative profile from one FACS run is shown. Counts indicate number of events (that is, cells detected). Ctrl, Control. **(C)** Western blot obtained after FOXM1 overexpression. **(D)** Expression of CD44 mRNA during exposure to Tamoxifen and the development of TamR cells. **(E)** FACS profile of MCF-7 and TamR cells with ABCG2-gated population. FSC-H, Forward scatter height. **(F)** ABCG2 expression levels during the time course of development of TamR cells. **(G)** Proliferation and 4-hydroxytamoxifen (OH-Tam) response with and without knockdown of ABCG2 in TamR cells. OD, Optical density. **(H)** Schematic representation of the separation of ABCG2+ and ABCG2- populations in TamR cells. FITC, Fluorescein isothiocyanate. **(I)** ABCG2+ and ABCG2- cell populations from TamR were sorted by FACS and monitored for proliferation in response to several concentrations of OH-Tam with and without FOXM1 knockdown. **(J)** FACS analysis of estrogen receptor α (ERα) levels in ABCG2+ and ABCG2- cell populations in TamR cells. **(K)** Percentage of Hoechst dye excluding side population (SP) cells in the overall TamR cell population or in ABCG2+ and ABCG2- cells after 12 days of culture. **(L)** Image of soft agar assay and three-dimensional Matrigel spheroid formation in TamR, ABCG2+ and ABCG2- cells. **(M)** Number of colonies formed in the soft agar assay and **(N)** Diameter of the colonies from TamR cells or separated ABCG2+ and ABCG2- cells. *P < 0.05; **P < 0.01.

GO analysis of the C1 cluster revealed enrichment for stem cell-related genes such as *ABCG2* and *SIRT1* and for the nuclear transcription factors *NF-YA* *,* *NF-YB* and *NF-YC*. On the basis of our bioinformatics analyses, we therefore hypothesized that the FOXM1 transcription program might play a role in the expansion of the CSC population and that this phenomenon might promote the acquisition of endocrine resistance. To validate this hypothesis, we first investigated the percentage of CSCs by FACS using CD44 and CD24 markers in MCF-7 parental cells, in siFOXM1 and in FOXM1-overexpressing MCF-7 cells, and in TamR cells. Interestingly, we observed that TamR cells had a fivefold higher percentage of CSCs compared to parental MCF-7 cells (Figure 5A). Furthermore, cells overexpressing FOXM1 had a CSC enrichment of about eightfold compared to parental cells (Figure 5A) and increased levels of *CD44* as shown by a FACS intensity plot (Figure 5B) and by a Western blot (Figure 5C). We also monitored the level of *CD44* mRNA during the development of endocrine resistance and observed that *CD44* mRNA increased with weeks of 4-OH-TAM exposure (Figure 5D). Thus, elevated FOXM1 is associated with increased expression of CSC markers.

Given recent reports describing ABCG2 as a marker of CSCs [50]-[52], and on the basis of our gene expression data, we compared the levels of ABCG2 in MCF-7 parental and TamR cells after FACS separation and observed a great enrichment (approximately 50-fold) in the ABCG2+ population in the TamR cells (Figure 5E). Moreover, ABCG2 mRNA levels increased progressively over time with TAM exposure, with the level increasing by about fivefold by 10 weeks and by approximately twelvefold by 100 weeks (Figure 5F). Next, to evaluate the functional role of FOXM1 in regulating the expression of ABCG2 and its involvement in TAM resistance, we reduced the level of ABCG2 by siRNA-mediated knockdown in TamR cells to 20% of the initial *ABCG2* mRNA level (Additional file 2: Figure S4) and then tested cell sensitivity to 4-OH-TAM. As shown in Figure 5G, 4-OH-TAM stimulated proliferation of the control TamR cells, whereas cells rendered deficient in ABCG2 became sensitive to growth suppression by 4-OH-TAM (Figure 5G).

Next, by FACS, we separated TamR cells based on the ABCG2 expression marker (Figure 5H) and evaluated cell proliferation in response to 4-OH-TAM in ABCG2+ and ABCG2- cells, with or without FOXM1 knockdown. Interestingly, 4-OH-TAM treatment in the control ABCG2+ cell population elicited growth stimulation, whereas FOXM1 knockdown in ABCG2+ cells prevented this growth-stimulatory effect (Figure 5I). Thus, the ABCG2+ population appears to contribute to the endocrine-resistant phenotype (Figure 5I). Because the ABCG2+ and ABCG2- cell populations express ERα mRNA at levels similar to each other and to those of the overall TamR cell population (Figure 5J), changes in ERα level do not explain their differences in proliferative response to TAM.

To examine the self-renewal properties of these cells, we sorted out ABCG2+ and ABCG2- TamR cells and cultured the different populations for 12 days. Because one of the defining characteristics of CSCs is their ability to transport Hoechst dye, which is attributable to expression of ABCG2, we examined Hoechst staining by FACS analysis. As shown in Figure 5K, ABCG2+ cells had a higher percentage of side population (SP) Hoechst-negative cells compared to the overall population of TamR cells or the ABCG2- cells.

Soft agar and three-dimensional Matrigel spheroid formation assays with the ABCG2+, ABCG2- and total TamR cell populations revealed that ABCG2+ cells not only had a more rapid growth rate compared to either TamR or ABCG2- cells (Figure 5L) but also formed more colonies (Figure 5M) and larger colonies (Figure 5N).

### FOXMoverexpression increases breast cancer cell aggressiveness and the expression of markers of epithelial-to-mesenchymal transition and cytoskeletal rearrangement

**Figure 6:**
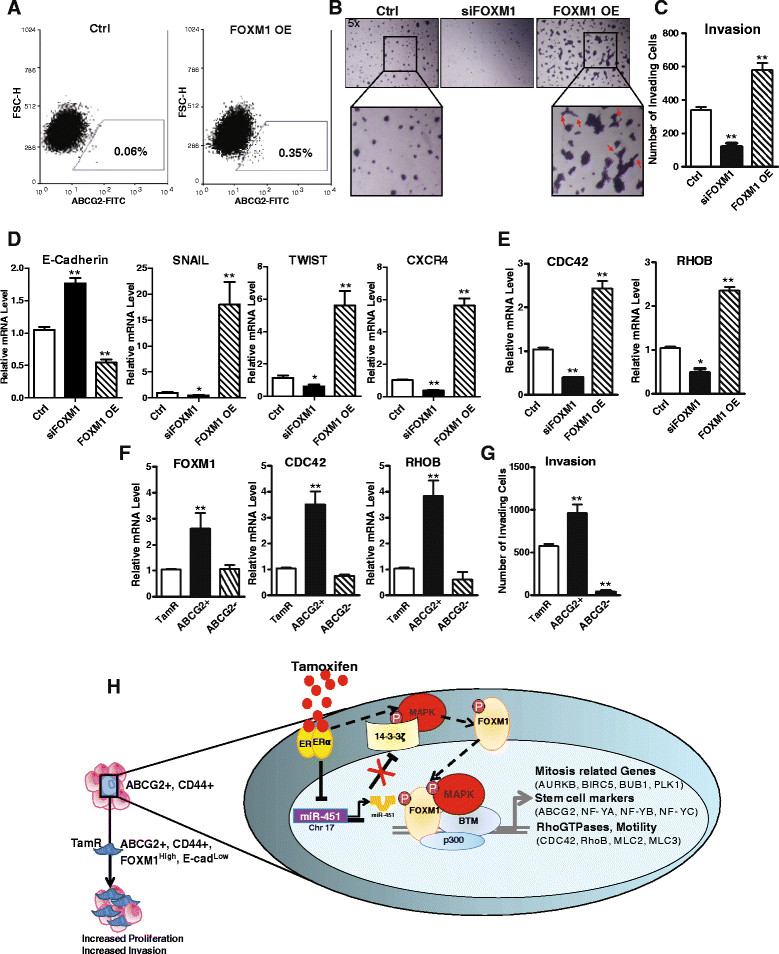
**FOXM1 increases expression of markers of epithelial-to-mesenchymal transition and invasiveness and induces an aggressive phenotype in breast cancer cells. (A)** Fluorescence-activated cell sorting (FACS) evaluation of the expression of ABCG2 in control (Ctrl) MCF-7 and FOXM1-overexpressing (OE) MCF-7 cells. A FACS profile from one of three representative experiments is shown. FITC, Fluorescein isothiocyanate; FSC-H, Forward scatter height. **(B)** Representative images obtained using a conventional inverted microscope show spheroids formed after modulation of the levels of FOXM1. Higher-magnification section (inset) shows details of invadopodia advancing into the matrix. **(C)** Invasion assay in Ctrl, siFOXM1 and FOXM1-OE MCF-7 cells after 48 hours. **(D)** Evaluation by quantitative RT-PCR (qRT-PCR) of the expression profiles of epithelial-to-mesenchymal transition markers and **(E)** Rho-GTPase genes CDC42 and RhoB in Ctrl or siFOXM1- or FOXM1-OE MCF-7 cells. Mean ± SEM. *p < 0.05; **p < 0.01 vs. Ctrl. **(F)** Evaluation by qRT-PCR of the expression profiles of FOXM1*,* CDC42 and RhoB in total TamR or ABCG2+ or ABCG2- cell populations. **(G)** Invasion assay in TamR and in sorted ABCG2+ and ABCG2- cells. Data are mean ± SEM. *P < 0.05; **P < 0.01 vs. total TamR cells. **(H)** Schematic model depicting our findings for the role of FOXM1 in engendering tamoxifen resistance, increased proliferation and invasion and the upregulation of stem cell markers, Rho-GTPases and mitosis-related genes. ER, Estrogen receptor; MAPK, Mitogen-activated protein kinase.

We observed a significant increase in the ABCG2+ population after overexpression of FOXM1 in MCF-7 cells for 72 hours, as examined by FACS (Figure 6A). Through evaluation of the functional role of FOXM1 in these cells by knockdown and overexpression, different phenotypes were clearly observed in three-dimensional Matrigel by as soon as 6 days (Figure 6B). No colony formation was seen in the FOXM1-knockdown (siFOXM1) population, whereas MCF-7 parental cells developed dense, round colonies. Cells overexpressing FOXM1 showed a very distinct phenotype characterized by branching chains of cells and cellular protrusions indicative of a migratory phenotype (Figure 6B). We also examined cell invasiveness using Boyden chambers and confirmed that FOXM1 overexpression engendered a more invasive phenotype, whereas invasion was decreased by FOXM1 reduction (Figure 6C).

The invasion process is associated with increases in EMT markers and loss of E-cadherin and requires cytoskeletal rearrangement. Hence, it was of interest that altering the level of FOXM1 modulated EMT markers *SNAIL, TWIST, CXCR4* and *E-cadherin* (Figure 6D). Further, our binding site cluster analysis revealed an enrichment of FOXM1 and ERα co-occupancy on genes involved in cytoskeleton regulation and rearrangement. These include *Rho-GTPase, CDC42* and *RhoB*. We observed that the expression of these genes was critically dependent on FOXM1 and was markedly altered by changing the level of FOXM1 (Figure 6E). We made similar observations in another ER+ cell line, T47D—namely, an increase in TAM-regulated growth suppression and a decrease in *ABCG2, CDC42* and *RhoB* gene and protein expression, as well as in pMAPK level, after treatment of the cells with the FOXM1 inhibitor ARF (Additional file 2: Figure S3).

As seen in Figure 6F, when we compared the levels of *FOXM1, CDC42* and *RhoB* in the sorted ABCG2+ and ABCG2- populations and in the total TamR cells, we consistently observed higher expression of *FOXM1* and Rho-GTPase genes in ABCG2+ cells (Figure 6F). Consonant with this, ABCG2+ cells had higher invasion capability compared to ABCG2- cells or the total TamR population (Figure 5G). Hence, the subpopulation of ABCG2+ cells within the overall TamR cell population is responsible for the more invasive phenotype of the TamR cells.

## Discussion

Our findings reveal that TAM resistance is associated with upregulation of FOXM1 and with a FOXM1-dependent gene expression program that enhances cell proliferation and invasiveness and elicits an increase in the proportion of CSCs within the breast cancer cell population. These cells expressed many markers associated with stem cells and with decreased patient survival [26], including CD44+ and CD24-/low markers, and elevated EMT markers and properties. They also showed high expression of ABC transporters that can result in tumor stem-like cells being resistant to conventional therapies due to drug efflux. These observations provide guidance for how one might optimally combine agents targeting specific characteristics of CSCs with conventional treatments that reduce tumor bulk, thereby effecting long-term benefits of ablating not only the overwhelming majority of the differentiated tumor cells but also removal of the more endocrine-resistant CSCs that can result in repopulation of the tumor [53]-[56]. Indeed, inherent drug resistance of CSCs is considered to be a crucial limitation to treatment effectiveness [56].

We found that the CSCs represent only a small proportion of the MCF-7 cell population, but that this fraction is increased fivefold in TamR cells. Our observations uncover a novel role for FOXM1 in inducing expansion of the CSC-like population and in promoting an aggressive and endocrine-resistant phenotype. These effects of FOXM1 likely underlie the strong association we have observed between high tumor FOXM1 and poor clinical outcome for patients with ER+ breast cancers. Our examination of several large data sets cumulatively representing about 1,000 ER+ breast tumors indicates that high FOXM1 expression occurs in about 20% of ER+ breast cancers. Of note, the authors of a recent report showed that FOXM1 and its regulated target genes *AURKA, AURKB* and *BIRC5/survivin* display the greatest prognostic discrimination among a panel of genes analyzed for overall survival of patients with ER+ breast cancer and an intermediate Oncotype DX 21-gene recurrence score. High expression of these genes predicts a poorer outcome and suggests more aggressive selection of adjuvant chemotherapy for these patients [57].

As schematized in the model (Figure 6H), we show that FOXM1 is elevated by TAM in a time-dependent manner and that its expression is associated with markers of TAM resistance. In previous studies, we identified the association between FOXM1 and 14-3-3ζ, a protein also found to be upregulated by TAM and elevated in TAM-resistant tumors [13] via deregulation of miR-451 that targets 14-3-3ζ [42] (Figure 6H). Our data now reveal that FOXM1, a member of the family of forkhead transcription factors, fosters the enrichment of CSCs expressing stem cell markers (for example, ABCG2, NF-YA, NF-YB and NF-YC), mitosis-related genes and genes fostering invasiveness and motility (Rho-GTPases).

By ChIP-Seq and ChIP-reChIP, we show that TAM induced recruitment of FOXM1 to the promoter regions of cell-cycle mitosis-related genes and genes encoding stem cell markers in MCF-7 and TamR cells, supporting our hypothesis that FOXM1 promotes the expansion of a highly proliferative CSC-like progenitor population that is capable of self-renewal and can give rise to differentiated progeny. We also observed FOXM1 upregulation of EMT markers.

We focused much of this study on our novel finding of the regulation by FOXM1 of stem cell–related genes that were found by seqMINER analysis to be enriched in the C1 cluster. Moreover, this cluster was also enriched for targets of miR-34a, recently reported to be important in regulating the expression of self-renewal genes [58]. Interestingly, the FOXM1 C1 cluster binding sites are co-occupied by ERK2, suggesting a sophisticated mechanism by which FOXM1 and MAPK signaling may participate in the development of endocrine resistance. Indeed, resistance to endocrine therapies is known to be associated with enhanced signaling through MAPK [1]-[3],[5],[8],[45]. We show in this study that the inhibition of MAPK activation with MEK1 inhibitor, or alteration of FOXM1 expression by the specific inhibitor ARF, impaired the recruitment of these factors to chromatin, indicating that these two factors control each other's binding to C1 genomic regions and that their copresence is essential for the activation of transcription of C1 genes. Of note, it has been shown that pMAPK induces phosphorylation of FOXM1, enabling its translocation to the nucleus and binding to genomic elements [59].

Further, our study reveals the interdependence of FOXM1 and MAPKs, with FOXM1 regulating the expression of MAPK and FOXM1-knockdown decreasing the level of MAPK. Of interest, the binding sites co-occupied by FOXM1 and MAPK are highly conserved among species, which suggests an evolutionarily conserved function for these genomic locations in different organisms. Moreover, our bioinformatics analysis of cluster C1 FOXM1-regulated genes was predictive of clinical outcome in women with TAM-treated tumors. Among these genes, we found well-described FOXM1 target genes such as *B-Myb, c-Jun* and *c-Fos*, as well as important genes involved in stem cell maintenance. Among the genes classified as CSC markers, we found multidrug resistance proteins (*MDR1, ABCG5* and *ABCG2*), the nuclear transcription factor *NF-YA/B/C*[60] and *SIRT1*[61].

We concentrated in particular on studying the role of FOXM1 in regulating the expression of ABCG2 because ABCG2, also known as breast cancer resistance protein, belongs to the ATP-binding cassette family. A defining feature of CSCs is their ability to efflux Hoechst dye, leading to the identification of the SP that is associated with expression of the ABCG2 protein. Its expression has been found in several stem cell tissues, including lung and prostate cancer and glioblastoma [62],[63]. Breast cancer SP cells have a high drug efflux capacity owing to functional expression of ABC transporters such as ABCG2. Although the mechanism by which multidrug resistance genes work in inducing chemotherapy resistance has been described previously, a recent study has implicated multidrug resistance proteins in hormone resistance by showing that ABCG2 can efflux TAM [64]. These reports support what we observed upon knockdown of either FOXM1 or ABCG2. With the reduction in cellular FOXM1 or ABCG2, or by inhibition of FOXM1 using ARF peptide, we were able to restore growth suppression by TAM to TamR cells, indicating that the levels of ABCG2 impact treatment response and that the upregulation of ABCG2 by FOXM1 could provide an explanation for the development of TAM therapy resistance.

In line with previous reports, our data show that our ABCG2+ SP had higher invasiveness potential compared to ABCG2- cells upon examination by three-dimensional Matrigel culture and invasion assays. We further determined that this phenomenon is associated with their elevated expression of *CDC42* and *RhoB* genes, which harbor FOXM1 binding sites co-occupied by ERα.

Of note, we show that overexpression of FOXM1 induced a cell phenotype characterized by branching, extended chains of cells and cellular protrusions distinctive of a migratory phenotype and characterized by increased expression of *CDC42* and *RhoB* and higher invasiveness. The GTP-binding proteins RhoB and CDC42 regulate the organization and turnover of the cytoskeleton and cell-matrix adhesions, which are a crucial feature in the acquisition of an invasive phenotype and the development of metastasis [65],[66]. Further, in line with what has been previously reported, our data confirm the binding of FOXM1 to matrix metalloproteinases and VEGF [14] as well as the regulation of EMT markers, thereby associating FOXM1 at yet another level to the metastatic process [20].

## Conclusions

Collectively, our findings define FOXM1 as a master regulator of Rho-GTPase and stem cell marker expression and imply that reducing FOXM1 expression might be effective in blocking tumor progression in several critical ways: by decreasing the expression of mitosis-related genes, by reducing invasion potential and by diminishing the proportion of CSCs, thereby enhancing sensitivity to cancer therapeutic agents. Indeed, the authors of several recent reports have shown FOXM1 to be associated with resistance to chemotherapeutic agents [67] and resistance to radiation treatment [68].

Moreover, our functional work clearly shows that rendering the FOXM1 pathway inactive by RNAi knockdown or by use of the p19^ARF^ 26-44 peptide [49], a selective FOXM1 peptide inhibitor called ARF, was highly effective in restoring endocrine sensitivity and suppressing breast cancer aggressiveness. This ARF inhibitor has already been shown to be effective in suppressing the development of hepatocellular carcinoma in a preclinical model [69]. Taken together, our findings have clinical implications for breast cancer and potentially many other cancers where FOXM1/pMAPK signaling pathways are active, and make a case for the use of FOXM1 inhibitors in combination with current therapies, including protein kinase inhibitors, to improve effectiveness and long-term patient response to treatments.

## Additional files

## Electronic supplementary material

Additional file 1: BED file detailing the FOXM1 and ERK2 binding regions.(XLSX 379 KB)

Additional file 2: Supplementary figures and figure legends.(PDF 11 MB)

Below are the links to the authors’ original submitted files for images.

Authors’ original file for figure 1

Authors’ original file for figure 2

Authors’ original file for figure 3

Authors’ original file for figure 4

Authors’ original file for figure 5

Authors’ original file for figure 6

## Abbreviations

EMT: Epithelial-to-mesenchymal transition
ERK2: Extracellular signal-regulated kinase 2
ERα: Estrogen receptor α
PBS: Phosphate-buffered saline
siRNA: Small interfering RNA
TMA: Tissue microarray
