# Supplementary material for: The forkhead transcription factor FOXM1 promotes endocrine resistance and invasiveness in estrogen receptor-positive breast cancer by expansion of stem-like cancer cells
Source: Breast Cancer Res. 2014 Sep 12;16:436. doi: 10.1186/s13058-014-0436-4 (PMC4303117; doi:10.1186/s13058-014-0436-4)

FOXM1 Promotes Endocrine Resistance and Invasiveness in Estrogen Receptor-Positive Breast Cancer by Expansion of Stem-Like Cancer Cells

Anna Bergamaschi, Zeynep Madak-Erdogan, Yu Jin Kim, Yoon-La Choi, Hailing Lu, and Benita S. Katzenellenbogen

Supplementary Figures

Fig. S1 Comparison of the binding sites for FOXM1, ERK2, and ERα after MCF-7 cell treatment for 45 min with Vehicle (0.1% ethanol), or 10^{-6} M Tam, or 10^{-8} M E2. SeqMiner clustering software was used based on co-occupancy of the different factors within a 600-bp window. All data are from our ChIP-seq studies, except for ERα binding site data in the presence of Tam which is from Hurtado et al. [1].

Fig. S2 Comparison of FOXM1 binding sites from MCF-7 cells after treatment with 10^{-6} M OH-Tamoxifen (this study) or from MCF-7 cells in media containing 10% fetal bovine serum and no added hormone or hormone antagonist [2]. Overlap of binding sites was calculated using Venn Diagram utility of Galaxy Cistrome using the default options of the program. 55% of the FOXM1 binding sites we identified after Tamoxifen were also found by Sanders et al. [2].

Fig. S3 Comparative analysis of FOXM1 and ARF regulation in the ER-positive T47D breast cancer cell line. A) Western blot showing FOXM1, ABCG2, CDC42 and pMAPK levels after ARF (FOXM1 inhibitor) or control (mutant ARF) treatment. **B**) Proliferation assay in T47D control (Ctrl) and ARF treated cells. **C**) OH-Tam sensitivity assay after ARF or control treatment of T47D cells. **D**) Evaluation by qPCR of the expression profiles of *FOXM1, ABCG2, CDC42* and *RhoB* genes after ARF inhibitor or control mutARF treatment. *, $p<0.05$, ** $p<0.01$ versus control.

Fig. S4 Representative photomicrographs showing each of the FOXM1 scoring categories (0, 1, 2, 3) for FOXM1 protein expression by immunohistochemical detection in breast tumor samples.

Fig. S5 Marked reduction of ABCG2 in Tamoxifen resistant MCF-7 (TamR) cells after treatment with ABCG2 siRNA. Cells were incubated with 20 nM control siRNA or ABCG2-siRNA for 72h and ABCG2 mRNA was then monitored by q-PCR. **, p<0.01.

References for Supplementary Information

1. Hurtado A, Holmes KA, Ross-Innes CS, Schmidt D, Carroll JS: **FOXA1 is a key determinant of estrogen receptor function and endocrine response.** *Nat Genet* 2011, 43(1):27-33.
2. Sanders DA, Ross-Innes CS, Beraldi D, Carroll JS, Balasubramanian S: **Genome-wide mapping of FOXM1 binding reveals co binding with estrogen receptor alpha in breast cancer cells.** *Genome Biol* 2013, 14(1):R6.

Supplementary Table S1. BED files of ChIP-seq data for FOXM1 and ERK2 binding sites in OH-tamoxifen-treated (10^{-6} M, 45 min) cells

Fig. S1

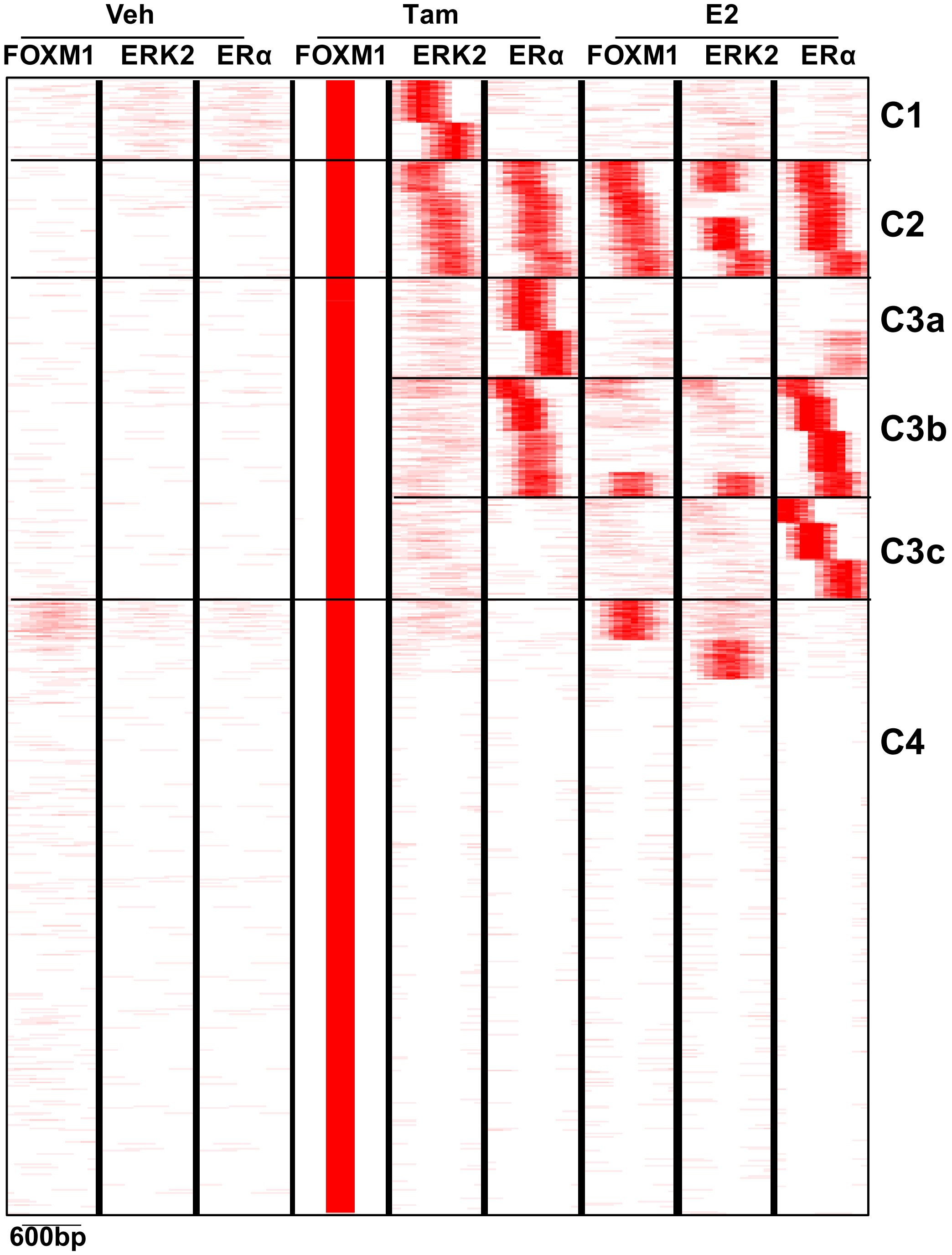
Veh
Tam
E2
FOXM1
ERK2
ERα
FOXM1
ERK2
ERα
FOXM1
ERK2
ERα
C1
C2
C3a
C3b
C3c
C4
600bp

Figure S2

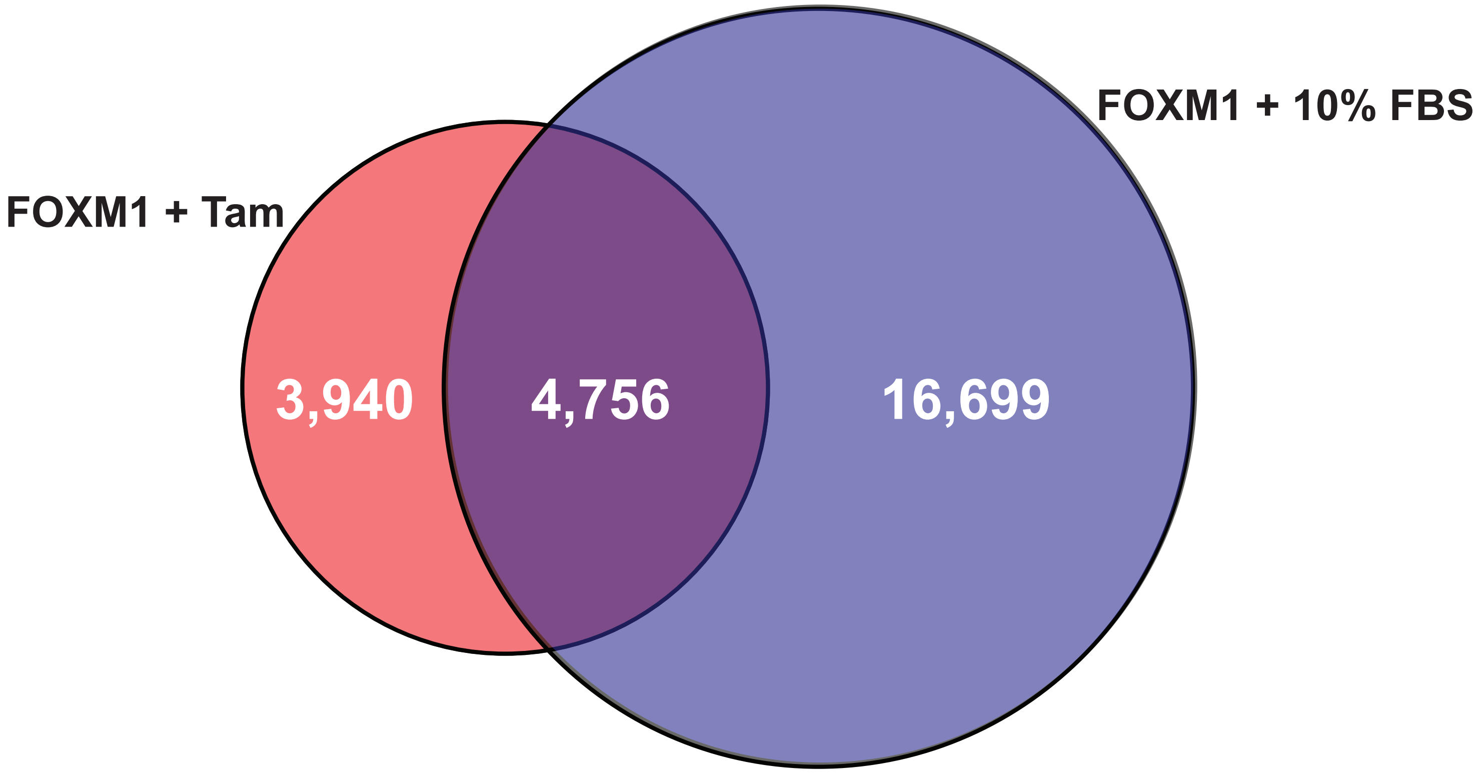

Fig. S3

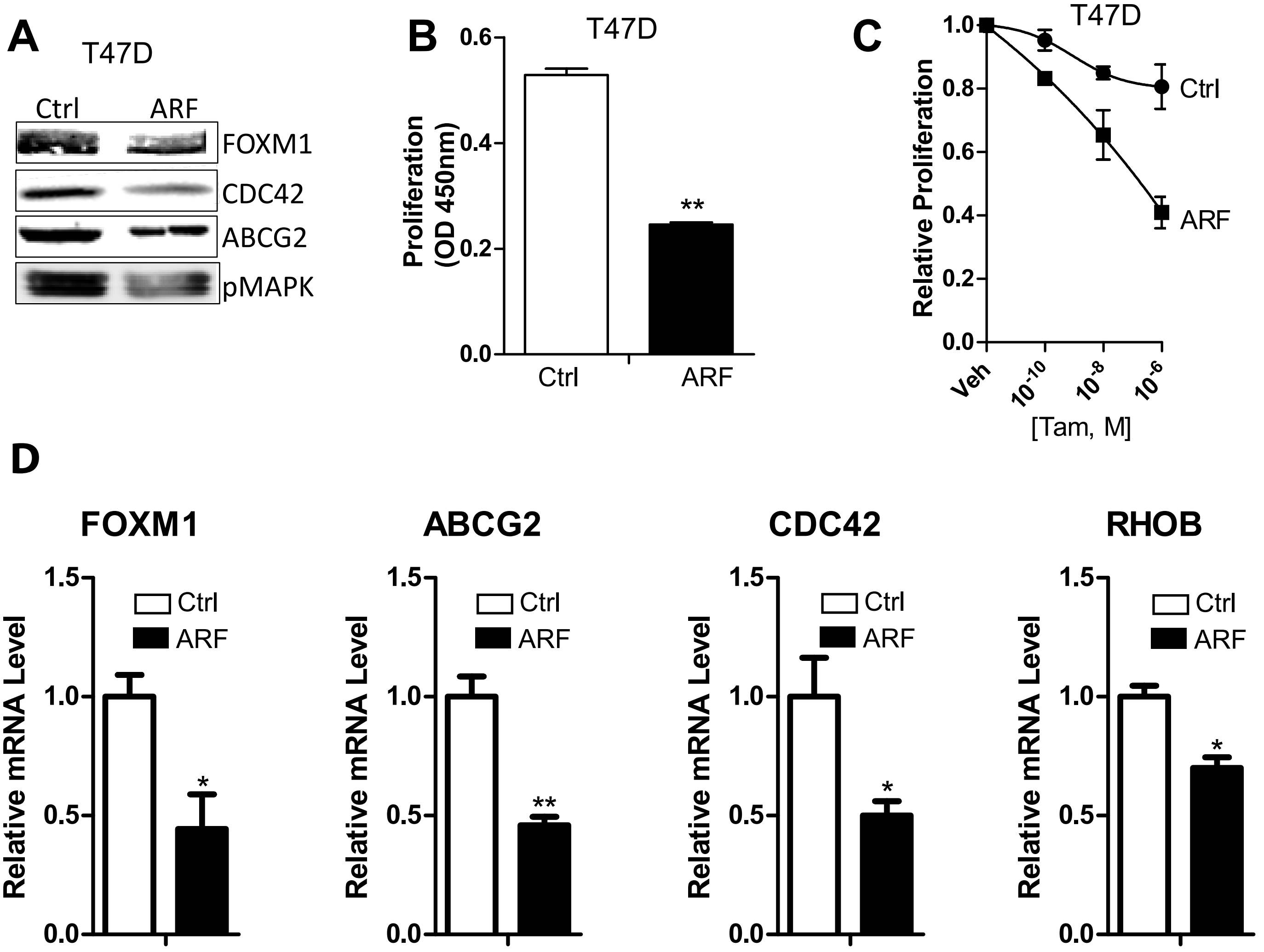
A
T47D
Ctrl
ARF
FOXM1
CDC42
ABCG2
pMAPK
B
T47D
Proliferation (OD 450nm)
0.6
0.4
0.2
0.0
**
Ctrl
ARF
C
T47D
Relative Proliferation
1.0
0.8
0.6
0.4
0.2
0.0
Ctrl
ARF
Veh
10-10
10-8
10-6
[Tam, M]
D
FOXM1
ABCG2
CDC42
RHOB
Relative mRNA Level
1.5
1.0
0.5
0.0
Ctrl
ARF
*
**
*
*

Figure S4

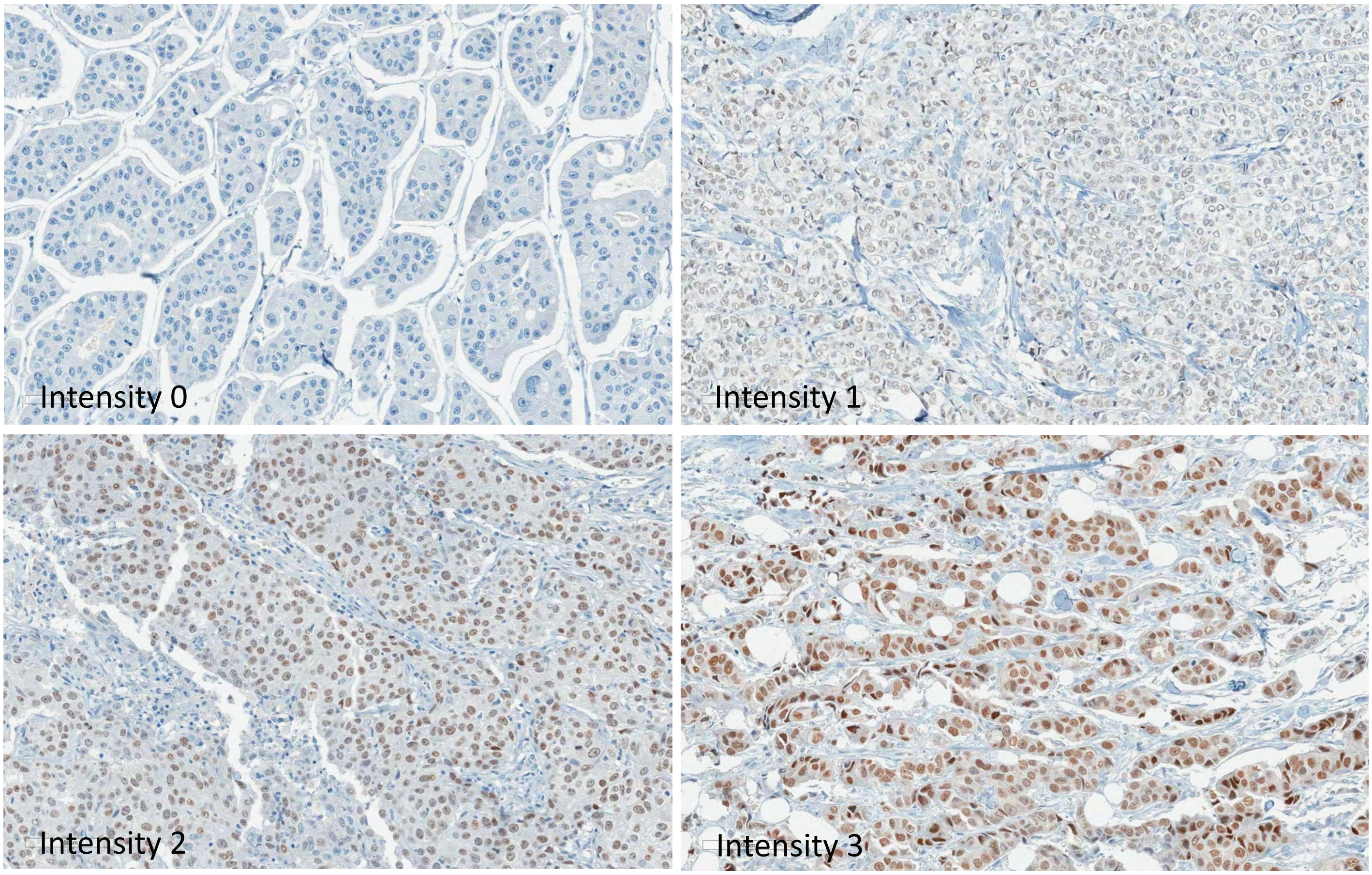

Figure S5

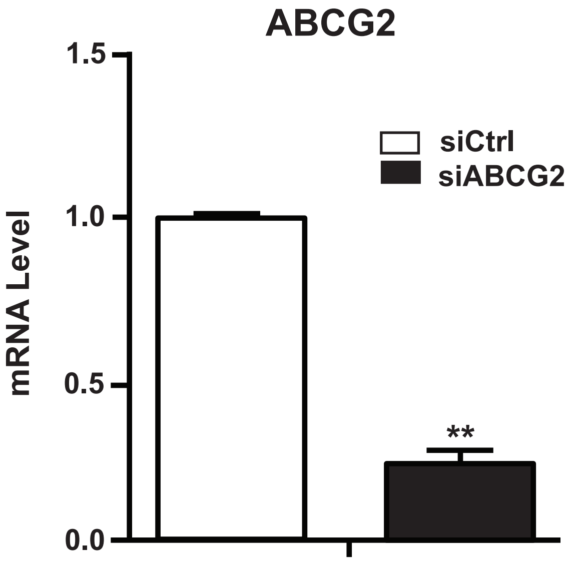

Supplement: Supplementary file 2 — Additional file 2: Supplementary figures and figure legends.(PDF 11 MB) [file 13058_2014_436_MOESM2_ESM.pdf]
