# Supplementary figures and images for: The forkhead transcription factor FOXM1 promotes endocrine resistance and invasiveness in estrogen receptor-positive breast cancer by expansion of stem-like cancer cells
Source: Breast Cancer Res. 2014 Sep 12;16:436. doi: 10.1186/s13058-014-0436-4 (PMC4303117; doi:10.1186/s13058-014-0436-4)

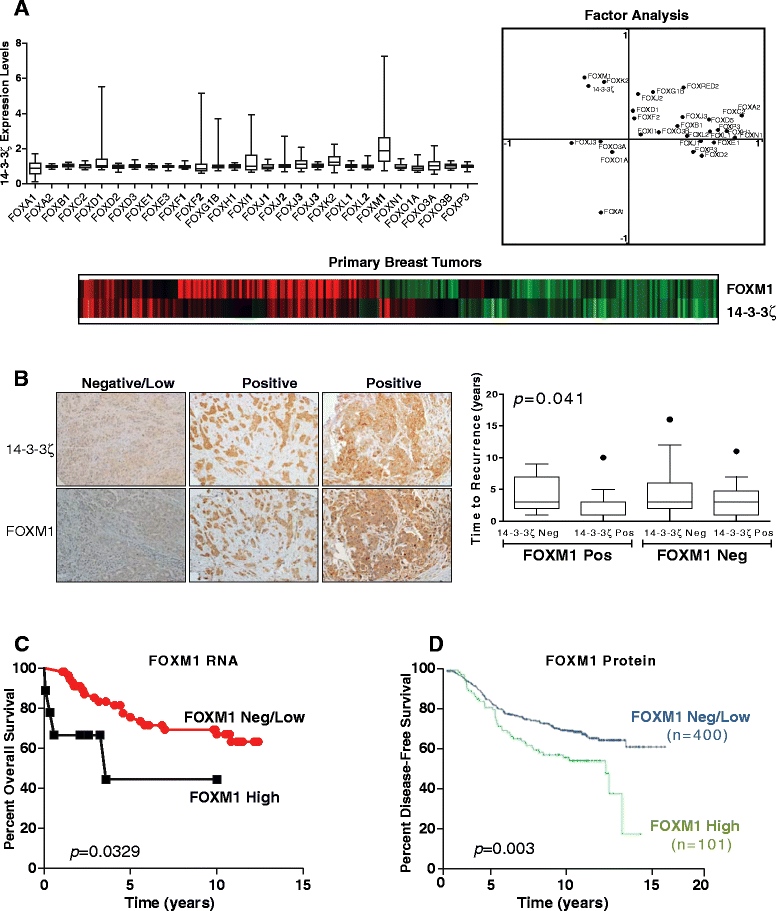

Supplement: Supplementary file 3 — Authors’ original file for figure 1 [file 13058_2014_436_MOESM3_ESM.gif]

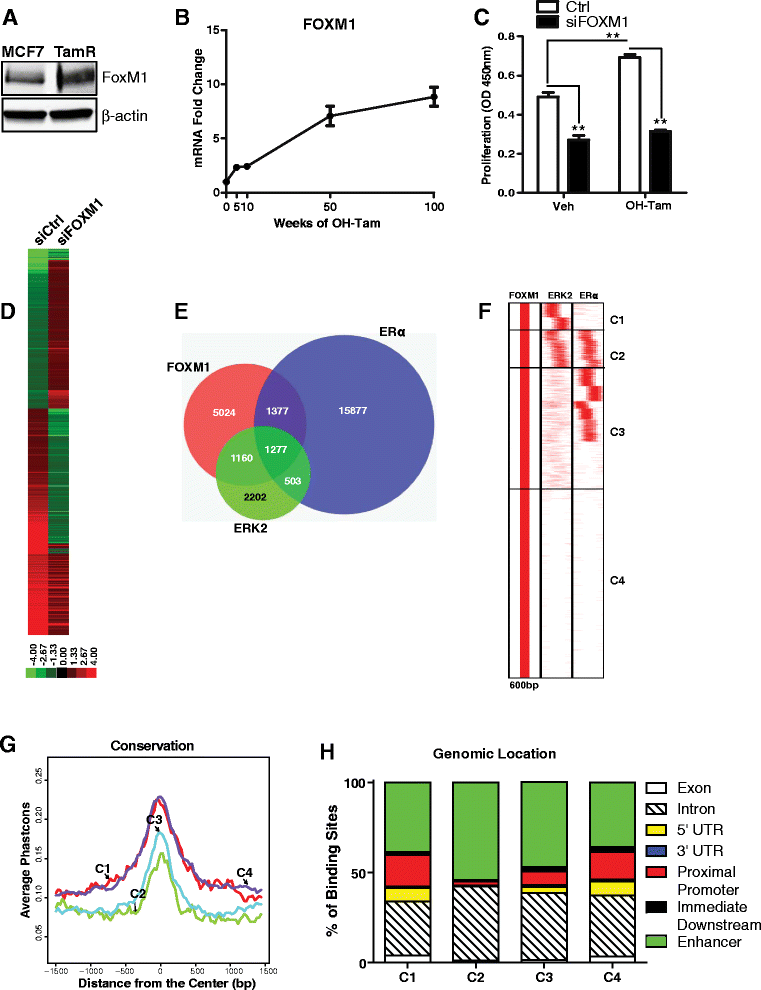

Supplement: Supplementary file 4 — Authors’ original file for figure 2 [file 13058_2014_436_MOESM4_ESM.gif]

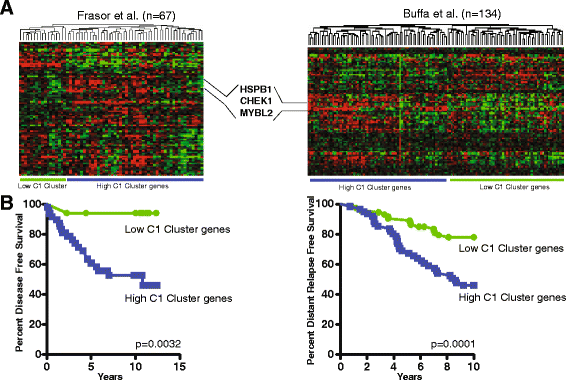

Supplement: Supplementary file 5 — Authors’ original file for figure 3 [file 13058_2014_436_MOESM5_ESM.gif]

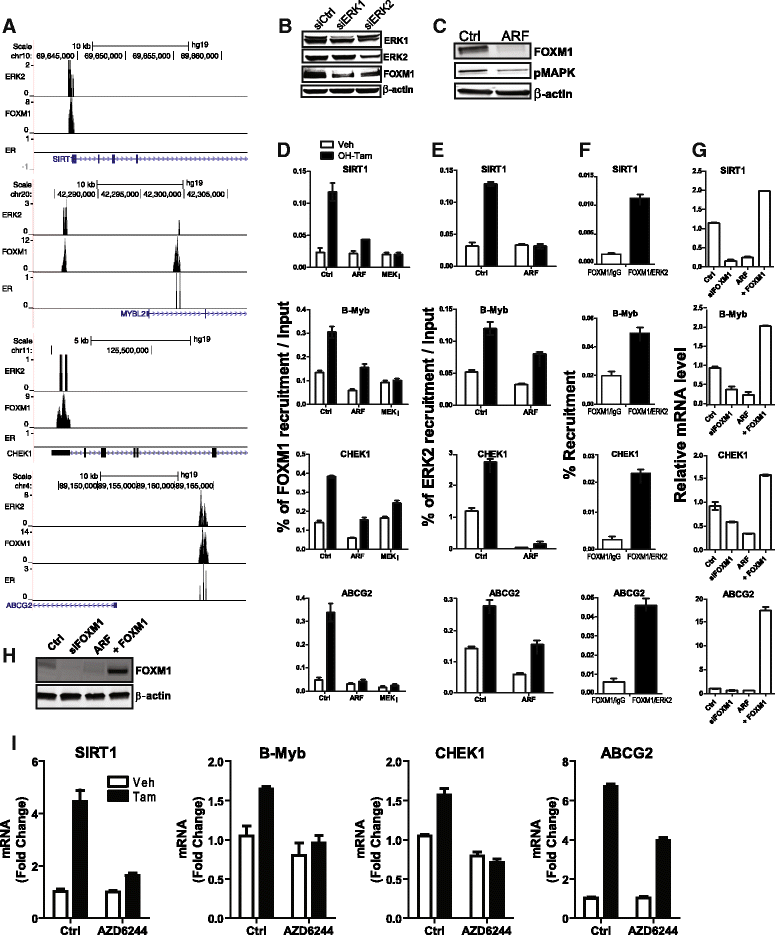

Supplement: Supplementary file 6 — Authors’ original file for figure 4 [file 13058_2014_436_MOESM6_ESM.gif]

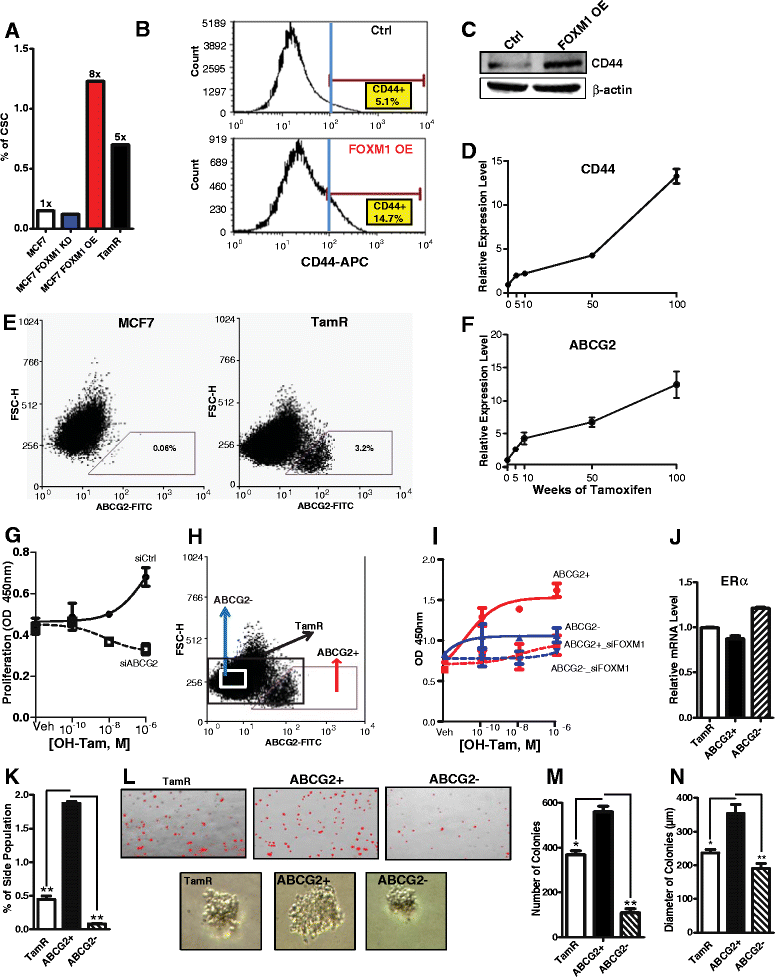

Supplement: Supplementary file 7 — Authors’ original file for figure 5 [file 13058_2014_436_MOESM7_ESM.gif]

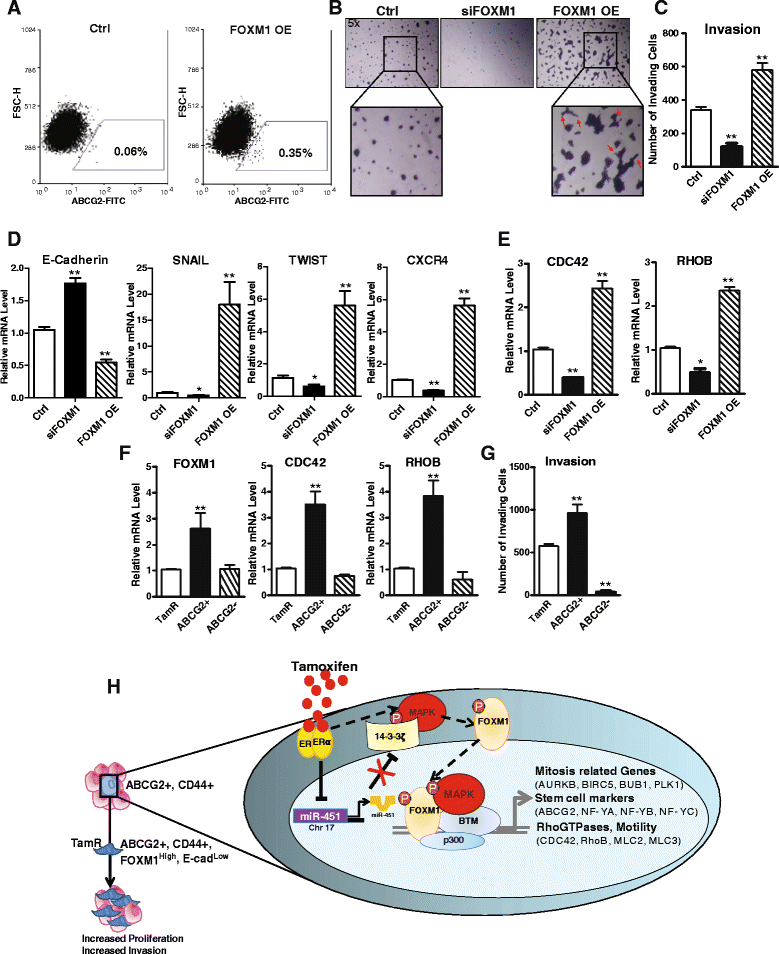

Supplement: Supplementary file 8 — Authors’ original file for figure 6 [file 13058_2014_436_MOESM8_ESM.gif]
